# Bruch’s membrane opening enlargement and its implication on the myopic optic nerve head

**DOI:** 10.1038/s41598-019-55926-w

**Published:** 2019-12-20

**Authors:** Mi Sun Sung, Min Young Heo, Hwan Heo, Sang Woo Park

**Affiliations:** Department of Ophthalmology and Research Institute of Medical Sciences, Chonnam National University Medical School and Hospital, Gwangju, South Korea

**Keywords:** Optic nerve diseases, Refractive errors

## Abstract

We examined the association between Bruch’s membrane opening (BMO) area and various ocular parameters and investigated the implication of BMO enlargement on the myopic optic nerve head. One hundred eighty-five myopic eyes were included in this cross-sectional study. Among the included eyes, 53 having axial lengths between 26 and 27 mm were further analyzed to investigate the association between BMO area and various ocular parameters. BMO area, BMO-minimum rim width (BMO-MRW), peripapillary choroidal thickness (pCT), width of β-parapapillary atrophy with and without Bruch’s membrane (PPA_+BM_ and PPA_−BM_), and presence of lamina cribrosa (LC) defect were evaluated. We found that BMO area tended to increase with increasing axial length, but varied among the highly myopic eyes even though they had similar degrees of myopia. In the subgroup analysis of eyes with axial lengths between 26 and 27 mm, BMO area was highly variable and it significantly correlated with PPA_−BM_ width and temporal-inferior, nasal-inferior, and nasal BMO-MRW and pCT. LC defects were more common in myopic eyes with enlarged BMO. A multivariate regression model revealed that higher intraocular pressure, enlarged BMO, and thinner BMO-MRW were associated with LC defects in highly myopic eyes. These findings should be considered when evaluating myopic eyes.

## Introduction

Histologically, the optic nerve head (ONH) is a three-layered opening through which the axons of the retinal ganglion cells (RGCs) pass; the innermost layer is Bruch’s membrane opening (BMO), the middle layer is the choroidal opening, and the third layer is the scleral canal opening^1–3^. Among the three openings, BMO is a clearly identifiable anatomical structure on spectral-domain optical coherence tomography (SD-OCT) and is thought to remain stable over time; thus, BMO is considered an anatomically more accurate and reliable landmark than is the conventional clinical disc margin when evaluating glaucoma^4,5^. Recently, BMO-based minimum rim width (MRW) measurement has become a new standard for documenting neuroretinal rim dimensions. Previous studies have reported that it has higher diagnostic accuracy for glaucoma and stronger relationship with visual field (VF) damage than do the conventional rim parameters^6–8^.

Myopic eyes demonstrate various morphological changes in the ONH^9,10^. Since the majority of structural diagnostic tools used to detect glaucoma are based on ONH evaluation, glaucoma diagnosis is difficult in myopic individuals with ONH deformation. In these patients, BMO-based parameters (e.g., BMO-MRW or BMO-based circumpapillary retinal nerve fiber layer [RNFL] thickness) have been proposed as good alternative diagnostic options^11–13^. Lee *et al*.^11^ suggested that BMO-based RNFL analysis is particularly advantageous in myopic eyes with a tilted optic disc. However, the clinical relevance of BMO-based parameters in myopic eyes is questionable, because the effect of axial elongation on BMO is not fully understood.

There are conflicting results on the association between BMO area and myopia. Some studies reported a positive correlation between BMO area and axial length^14,15^. In contrast, a study on a normal Caucasian population reported that BMO area was not significantly associated with axial length^16^. Moreover, in a recent prospective and longitudinal observational study, Kim *et al*.^17^ showed that the distance between two BMO points on horizontal OCT B-scan images remained stable during myopia progression. Therefore, the purpose of this study were to determine whether BMO area is affected by axial elongation and to examine the association between BMO area and various ocular parameters in myopic eyes. Additionally, we tried to elucidate the implication of BMO enlargement on the myopic ONH.

## Methods

### Subjects

Study subjects were recruited from the Young Myopia Study of Chonnam National University Hospital, which is an ongoing cross-sectional study that commenced in January 2018. The present study was approved by the Institutional Review Board of Chonnam National University Hospital and it adhered to the tenets of the Declaration of Helsinki. All patients provided written informed consents prior to enrollment in the study.

The Young Myopia Study enrolled consecutive participants who visited the general eye clinic for medical check-up and met all of the inclusion criteria and none of the exclusion criteria. All subjects underwent comprehensive ophthalmologic examination consisting of the measurement of best-corrected visual acuity (BCVA), intraocular pressure (IOP) by Goldmann applanation tonometry, and refractive error by automated refraction. Anterior chamber angle assessment using gonioscopy was performed on all eyes. ONH and RNFL examination using color stereoscopic disc photography and red-free RNFL fundus photography, and Swedish Interactive Threshold Algorithm standard 30-2 perimetry with a Humphrey Field Analyzer (Carl Zeiss Meditec Inc., Dublin, CA, USA) were performed. Axial length, central corneal thickness, and corneal curvature were measured using optical low-coherence reflectometry (Lenstar; Haag-Streit AG, Koeniz, Switzerland). For all subjects, a detailed medical history was recorded.

The following inclusion criteria were used: (1) healthy subjects aged between 20 and 35 years, (2) a spherical equivalent (SE) refractive error between −0.5 and −12 diopters (D), (3) a cylinder correction of −2.0 to +2.0 D, (4) BCVA ≥ 20/25, (5) IOP ≤ 21 mmHg, (6) normal anterior chamber angles, (7) nonglaucomatous ONHs on disc photographs (an intact neuroretinal rim without peripapillary hemorrhage, thinning, or localized pallor), (8) absence of any RNFL abnormalities on red-free fundus photographs, and (9) normal VF (defined as a glaucoma hemifield test within normal limits and a pattern standard deviation within 95% confidence-interval limits) results in both eyes. We excluded subjects older than 35 years in this study, since the lenticular changes can affect refractive error, and aging itself may increase the incidence of glaucoma. Eyes having an enlarged blind spot associated with a large area of parapapillary atrophy (PPA) were also included in this study.

Subjects were excluded if they had any of the following: (1) a family history of glaucoma in a first-degree relative, (2) history of intraocular or refractive surgery, (3) pathologic myopia (patch chorioretinal atrophy, lacquer crack lesions, intrachoroidal cavitations, or choroidal neovascularization), (4) other evidence of retinal pathology, (5) opaque media, or (6) poor-quality OCT images because of irregular tear film or poor cooperation. Eligibility was determined by 2 glaucoma specialists (S.W.P and M.S.S), who evaluated optic disc appearance on stereoscopic disc photographs and RNFL defects on red-free fundus photographs. The evaluators were masked to the subject’s clinical status and ocular data, and an eye was excluded from study analyses if a consensus could not be reached. When both eyes of a patient were eligible for the study, 1 eye was randomly selected.

### Spectral-domain optical coherence tomography imaging

All participants underwent OCT imaging using SD-OCT (Heidelberg Spectralis SD-OCT; Spectralis software version 6.9.4; Heidelberg Engineering GmbH, Heidelberg, Germany). One experienced operator (M.Y.H) performed all OCT scans. Magnification error was corrected using the formula provided by the manufacturer on the basis of the results of keratometry and focus setting during image acquisition. Scan images with insufficient quality (typically truncated B-scans and quality score <30) were excluded.

**Figure 1:**
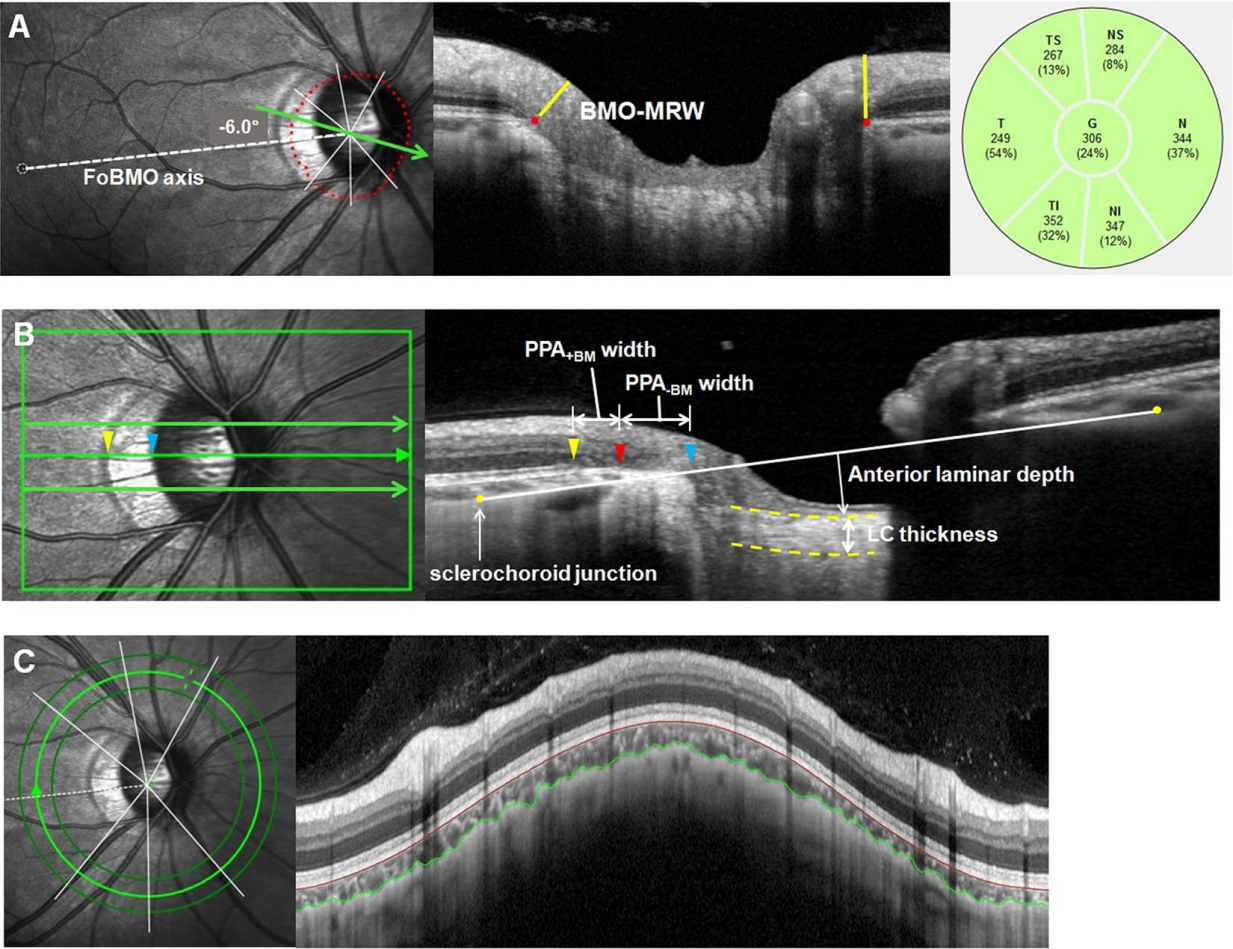
Methods used for ONH analysis by using SD-OCT. (**A**) Bruch’s membrane opening (red dots) is identified on confocal scanning laser ophthalmoscopy and B-scan images. FoBMO axis (the line connecting the center of the fovea and BMO center) and FoBMO angle are also illustrated on the fundus image. The distance between the termination of BM and the nearest point on the ILM in each B-scan image is defined as BMO-MRW. The global and the 6 Garway-Heath regional BMO-MRW values (NS, N, NI, TI, T, and TS) are calculated. (**B**) EDI OCT scan image passing through the ONH (center, midsuperior, and midinferior) is presented. The yellow arrow head indicates the RPE termination, red arrow head indicates the termination of BM, and blue arrow head indicates the optic disc margin. The distance from the edge of BM (red arrow head) to the margin of RPE termination (yellow arrow head) and to the optic disc margin (blue arrow head) are defined as PPA_+BM_ width and PPA_−BM_ width, respectively. Anterior laminar depth is defined as the distance between the sclerochoroid junction reference line (white solid line) and the anterior border of the LC (yellow dotted line). LC thickness is defined as the distance between the anterior and posterior borders (yellow dotted lines) of the LC. (**C**) Circular peripapillary scan of 4.1 mm is obtained. After the manual segmentation of BM and the sclerochoroidal border (green solid line), peripapillary choroidal thickness was calculated. BM = Bruch’s membrane; BMO = Bruch’s membrane opening; MRW = minimum rim width; RPE = retinal pigment epithelium; PPA_+BM_ = β-parapapillary atrophy with BM; PPA_−BM_ = β-parapapillary atrophy without BM; LC = lamina cribrosa.

BMO and BMO-MRW were measured using the Glaucoma Module Premium Edition (GMPE) software (Fig. 1A). The scan protocol composed of 24 equally spaced radial B-scans, each with 768 A-scans covering a 15° region centered on the optic disc. Twenty-five B-scans were averaged automatically for each scan location. The software automatically segmented the internal limiting membrane (ILM) and the 48 BMO points from the 24 radial scans. All of the B-scans were manually checked, and BMO points were corrected when necessary. When BMO points were indiscernible, the points were fitted with a spline to derive a closed curve and a smooth contour line based on BMO points of adjacent B-scans. If BMO points were indiscernible on 4 or more consecutive B-scan images, the eyes were excluded from the study. First, BMO points were checked by one experienced evaluator (M.S.S) and then reassessed and confirmed by a senior glaucoma specialist (S.W.P). In case of any discrepancy, the eyes were excluded from the analysis. BMO area was computed automatically by the built-in software. In this scan mode, the foveal pit and 2 BMO points in each of the 2 radial B-scans that were perpendicular to each other were automatically segmented to estimate the center of BMO and determine the FoBMO axis (the line connecting the center of the fovea and BMO center), which served as a reference for the scans. The FoBMO angle was defined as the angle between the FoBMO axis and the horizontal axis of the acquired image frame.

Additionally, the GMPE software measured the distance between the termination of Bruch’s membrane and the nearest point on the ILM in each B-scan image. The global and the 6 Garway-Heath regional BMO-MRW values relative to the FoBMO axis (nasal-superior [NS, 85–125°], nasal [N, 125–235°], nasal-inferior [NI, 235–275°], temporal-inferior [TI, 275–315°], temporal [T, 315–45°], and temporal-superior [TS, 45–85°]) were calculated.

### Optic nerve head measurements

The Heidelberg Spectralis OCT enhanced depth imaging (EDI) mode was used for the other ONH measurements (Fig. 1B). The ONH was scanned by centering a 15° × 10° rectangular scan on the ONH. Each OCT volume consisted of 49 serial horizontal B-scans (4.5-mm-long lines; 50 images averaged) spaced at approximately 63-μm intervals. Infrared (IR) fundus images were acquired simultaneously by using a confocal scanning laser ophthalmoscope. Three sections that passed through the ONH in the center, midsuperior, and midinferior regions were selected, and all the study parameters were measured in each of these frames by 2 independent examiners (M.S.S and H.H), in a masked fashion.

Temporal β-zone PPA margin and disc margin were defined using IR fundus images. Temporal BMO points were identified in the horizontal EDI scan image. PPA width was measured using the synchronous viewing of the IR fundus image and the selected location on the OCT scan. β-PPA width was defined as the distance between the beginning of the retinal pigment epithelium (RPE) (i.e., temporal β-PPA margin) and temporal disc margin on each horizontal B-scan image. On the basis of the location of BM termination, the β-PPA was further divided into PPA_+BM_ and PPA_−BM_ (γ-zone PPA); PPA_+BM_ width was defined as the distance from the beginning of the RPE to BM, and PPA_−BM_ was defined as the distance from the temporal disc margin to the beginning of BM. The measurement was performed using a previously described method^18–21^.

To overcome the effect of choroidal thickness on lamina cribrosa (LC) depth measurements, the sclerochoroid junction reference plane was used^22,23^. The sclerochoroid junction reference line was defined as the line connecting 2 points of the anterior scleral surface located at 1750 μm from the center of BMO in each B-scan image. The vertical distance between the reference line and the anterior LC surface was measured at the center of the ONH and defined as anterior laminar depth. The anterior and posterior margins of the highly reflective region at the ONH vertical center in each B-scan image were used as the borders of the LC, and the perpendicular distance between these 2 borders was defined as LC thickness. In cases where the central retinal vessel trunk prevented visualization, measurements were performed on the temporal side.

The measurement was performed using a built-in caliper tool of the intrinsic OCT viewer, and average data of 3 horizontal B-scan images (center, midsuperior, and midinferior) were calculated and used in this study. The mean of the values obtained by the 2 examiners was used in the final analysis.

### Detection of lamina cribrosa defect

The EDI OCT images of the ONH were reviewed carefully for detecting focal LC defects. A focal LC defect was defined as a loss of high reflectivity from the anterior-to-posterior border of the full-thickness LC on B-scan images. To avoid false-positives, the defects were required to have had a maximal diameter greater than 100 μm and a depth greater than 30 μm, and to have been present in two adjacent B-scans^24,25^. Shadows were differentiated from LC defects on the basis of their characteristic signal void behind the vessels and tissues. LC defect margins were independent of the location of the vessels and neural tissues. Example of LC defect is provided in Supplementary Fig. S1. Images were reviewed by 2 experienced glaucoma specialists (M.S.S and S.W.P), and disagreements were addressed via discussion between the 2 evaluators to achieve consensus. An eye was excluded from study analyses if a consensus could not be reached.

### Choroid thickness measurement

We obtained 360° circular RNFL measurements centered on BMO center. Among the 3 circular scans along the peripapillary circles (diameters of 3.5, 4.1, and 4.7 mm), the 4.1-mm-diameter scans were analyzed to minimize the interference of a large PPA on the OCT scan path. For the measurement of peripapillary choroidal thickness (pCT), the upper and lower segmentation lines of the circular scan were manually delineated. The lines were adjusted to align with the inner scleral wall and posterior border of the RPE to define the outer and inner boundaries of the choroid, respectively (Fig. 1C). pCT was automatically computed using the RNFL thickness sector algorithms (global, NS, N, NI, TI, T, and TS). Subfoveal choroidal thickness was manually assessed on the EDI-OCT scan images running through the fovea. The vertical distance from the outer edge of hyper-reflective RPE to the inner border of the sclera at the fovea was measured. Images in which the RPE and inner border of the sclera could not be clearly identified were excluded. The average of data from 2 independent evaluators (M.S.S and H.H) was used in the analysis.

### Statistical analysis

SPSS version 21.0 (SPSS, Chicago, IL, USA) and R version 3.3.1 were used for all statistical analyses. Agreement on PPA_+BM_ width, PPA_−BM_ width, LC thickness, anterior laminar depth, and peripapillary and subfoveal choroidal thickness between 2 observers was assessed using the Bland-Altman method, which plots the means against differences^26^. The limits of agreement were defined as the mean differences of 2 measurements ±1.96 standard deviation of the difference. The normality of distribution was verified using the Shapiro-Wilk normality test. Levene’s test was used to check the homogeneity of variance between the different axial length groups. The main parameters were presented as the counts and proportions or mean ± standard deviation values. Groups were compared using the chi-square test, Student’s *t* test, or Mann-Whitney *U* test as appropriate. Linear regressions were used to search for associations between BMO area and various ocular parameters. Further assessment of the association between BMO area and ocular parameters was performed after including age, sex, and axial length as covariates. Coefficients with 95% confidence intervals were presented. *P* values were adjusted to control the false discovery rate using the Benjamini-Hochberg procedure^27^. Factors associated with the presence of LC defects were assessed using multivariate logistic regression analysis. Confounders were identified using a threshold of *P* < 0.10 in the comparison of baseline characteristics. Then, all variables with a significance level of less than 0.10 were included in the multivariate model (enter method). Sectorial BMO-MRW values were not included in the multivariate analysis to reduce collinearity in the final model. A *P* value < 0.05 was considered statistically significant.

## Results

The study initially enrolled 205 eyes with myopia. Of these 8 eyes (3.90%) were excluded because of poor OCT image quality. Twelve eyes (5.85%) were additionally excluded because of indiscernible BMO on the 4 or more consecutive B-scan images or discrepancy in BMO determination between the glaucoma specialists (M.S.S and S.W.P). None of the subjects was excluded because of the disagreement on LC defect between specialists or inability to determine RPE and chorioscleral interface. Finally, 185 eyes of 205 myopic eyes were included in the analysis. Interobserver agreement, determined using Bland-Altman plots, in the measurements of PPA_+BM_ width, PPA_−BM_ width, LC thickness, and peripapillary and subfoveal choroidal thickness for all the subjects showed no systematic differences in measurements (data not shown).

**Figure 2:**
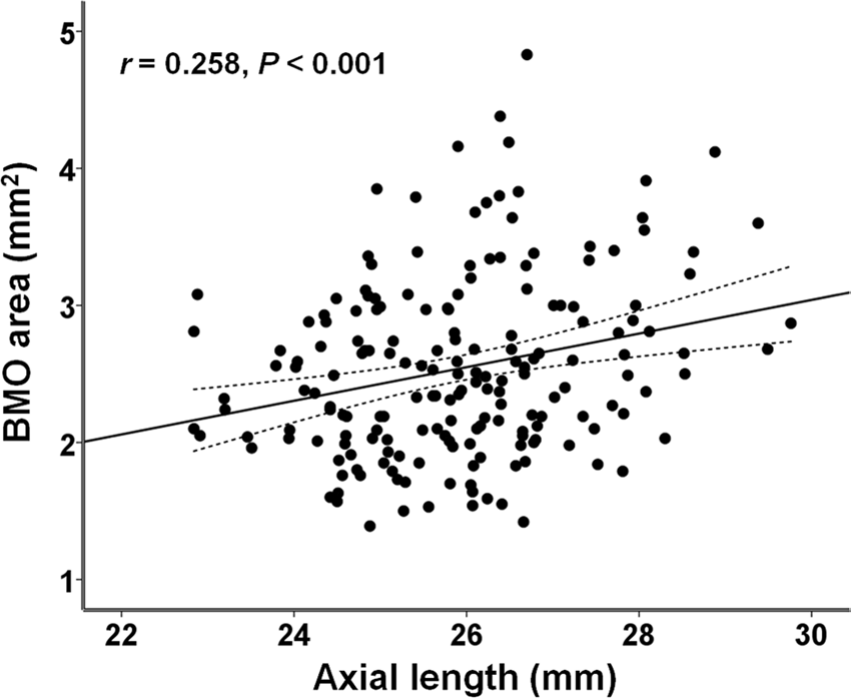
Distribution of BMO area values with axial length in 185 myopic subjects. The scatter plot shows that BMO area increases with increasing axial length. BMO = Bruch’s membrane opening.

**Figure 3:**
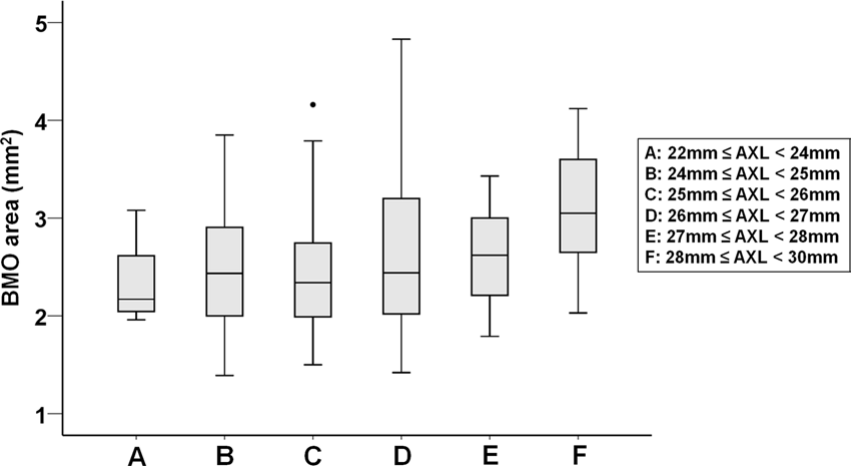
Box-and-whisker plots showing the distribution of BMO area according to the axial length group among 185 myopic eyes. BMO = Bruch’s membrane opening; AXL = axial length.

The cohort included 125 male (67.57%) and 60 female (32.43%) subjects with a mean age of 27.06 ± 2.77 years. The mean SE refractive error was −5.07 ± 2.90 D, mean axial length was 25.91 ± 1.37 mm, and BMO area was 2.54 ± 0.65 mm^2^. BMO area tended to increase with increasing axial length (*r* = 0.258, *P* < 0.001) (Fig. 2). When the eyes were divided according to their axial length into 6 groups (group A = 22 mm ≤ axial length <24 mm, n = 12; group B = 24 mm ≤ axial length <25 mm, n = 40; group C = 25 mm ≤ axial length <26 mm, n = 44; group D = 26 mm ≤ axial length <27 mm, n = 53; group E = 27 mm ≤ axial length <28 mm, n = 22; and group F = 28 mm ≤ axial length <30 mm, n = 14), mean SE refractive error was −0.17 ± 0.78 D, −3.02 ± 1.52 D, −4.57 ± 1.91 D, −5.62 ± 1.74 D, −7.85 ± 1.56 D, and −10.37 ± 1.67 D in group A, B, C, D, E, and F, respectively. When the homogeneity of variance of BMO area was assessed using Levene’s test, a significant difference in variance was observed between the groups (*P* = 0.027). The distribution of BMO area across the different axial length groups is illustrated as a box-and-whisker plot in Fig. 3. Generally, a larger standard deviation of BMO area values was noted in group D.

We next focused on how the ONH is affected by BMO enlargement in myopic eyes. Eyes in group D were chosen for further association analysis because the variability of BMO area values was generally more pronounced in that group. We thought that the effect of BMO enlargement on ONH characteristics might be observed more prominently in the group with high BMO area variability. In addition, by employing strict inclusion criteria for axial length, the influence of axial elongation could be minimized. Because axial elongation itself also could accompany various morphological changes of ONH. Eventually, 53 eyes having axial lengths between 26 and 27 mm were further analyzed for investigating the association between BMO area and various ocular parameters. A summary of the demographic variables and the results of the OCT measurements are shown in Table 1. Fifty-three eyes had a mean axial length of 26.43 ± 0.27 mm, and BMO area varied widely with a standard deviation of 0.80 mm^2^ (range, 1.42 mm^2^ to 4.83 mm^2^).

**Table 1:** Summary of subjects (26 mm ≤ axial length <27 mm) characteristics.

Variables	Description
Number, n	53
Age (years)	26.72 ± 2.41
Male, n (%)	35 (66.04)
SE refractive error (D)	−5.62 ± 1.74
Axial length (mm)	26.43 ± 0.27
Central corneal thickness (μm)	552.89 ± 27.64
Corneal curvature (D)	42.57 ± 1.06
IOP (mmHg)	13.98 ± 2.57
BMO area (mm^2^)	2.59 ± 0.80
PPA_+BM_ width (μm)	177.28 ± 179.62
PPA_−BM_ (γ zone PPA) width (μm)	369.43 ± 253.34
LC thickness (μm)	216.70 ± 38.78
Anterior laminar depth (μm)	330.75 ± 108.76
FoBMO angle (°)	9.86 ± 4.46
Presence of LC defect, n (%)	20 (37.74)
BMO-MRW (μm)	
Global	337.58 ± 44.85
Temporal-superior	338.87 ± 51.40
Temporal	242.01 ± 33.76
Temporal-inferior	355.72 ± 55.54
Nasal-inferior	389.67 ± 53.21
Nasal	371.51 ± 62.13
Nasal-superior	387.58 ± 79.95
Peripapillary choroidal thickness (μm)	
Global	146.88 ± 46.34
Temporal-superior	157.08 ± 56.78
Temporal	135.92 ± 52.75
Temporal-inferior	120.65 ± 42.75
Nasal-inferior	125.69 ± 43.55
Nasal	161.40 ± 49.98
Nasal-superior	168.98 ± 56.14
Subfoveal choroidal thickness (μm)	250.45 ± 83.02

SE = spherical equivalent; D = diopter; IOP = intraocular pressure; BMO = Bruch’s membrane opening; PPA_+BM_ = β-parapapillary atrophy with Bruch’s membrane; PPA_−BM_ = β-parapapillary atrophy without Bruch’s membrane; LC = lamina cribrosa; FoBMO angle = angle of fovea-to-BMO-center axis relative to the horizontal axis of the image frame; MRW = minimum rim width.

Data are mean ± standard deviation unless otherwise indicated.

**Figure 4:**
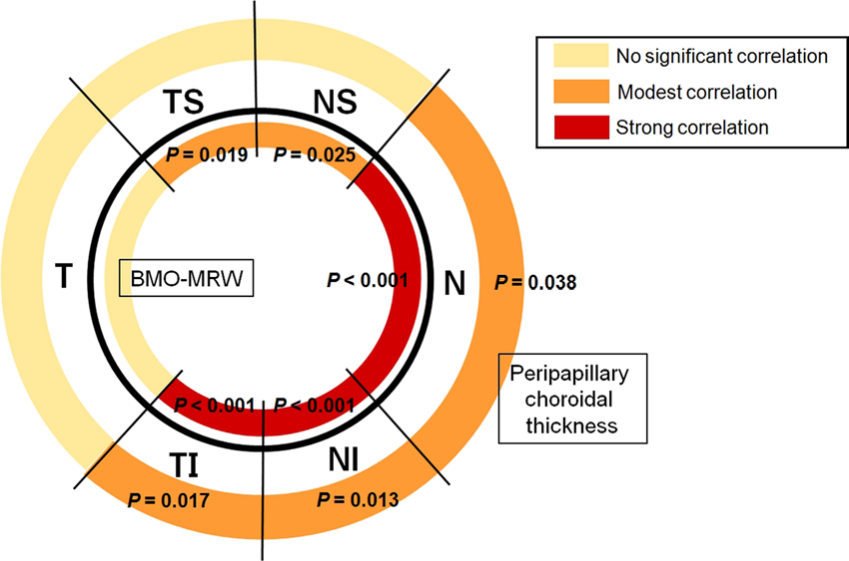
Diagram illustrating the association between BMO-MRW (inner color-coded circle) and peripapillary choroidal thickness (outer color-coded circle) and BMO area in 53 myopic eyes with axial length between 26 and 27 mm. *P* values shown are adjusted for age, sex, and axial length. BMO = Bruch’s membrane opening; MRW = minimum rim width; NS = nasal-superior; N = nasal; NI = nasal-inferior; TI = temporal-inferior; T = temporal; TS = temporal-superior.

A larger BMO area was significantly associated with a larger PPA_−BM_ width (*P* < 0.001), deeper anterior laminar depth (*P* = 0.031), and thinner global BMO-MRW (*P* < 0.001) and global pCT (*P* = 0.034). These associations remained significant even after adjusting for age, sex, and axial length (Table 2). Sectorially, the relationship between the BMO-MRW and BMO area varied notably, with a strong correlation in the temporal-inferior, nasal-inferior, and nasal BMO-MRW sectors (all *P* < 0.001, after adjusting for age, sex, and axial length) and a modest correlation in the temporal-superior and nasal-superior BMO-MRW sectors (*P* = 0.019 and *P* = 0.024, respectively, after adjusting for age, sex, and axial length). No significant association was found in the temporal BMO-MRW sector. The association between the corresponding sectorial pCT and BMO area showed a similar tendency as the results of the association between the sectorial BMO-MRW values and BMO area. BMO area correlated significantly with the temporal-inferior, nasal-inferior, and nasal peripapillary choroidal thicknesses (*P* = 0.017, *P* = 0.013, and *P* = 0.038, respectively, after adjusting for age, sex, and axial length). The associations between the sectorial BMO-MRW and pCT and BMO area are schematically illustrated in Fig. 4.

**Table 2:** Associations between the BMO area and various clinical and ocular parameters among the 53 subjects (26 mm ≤ axial length <27 mm).

Variables	Univariable		Adjusted for age, sex and axial length	
	Coefficient (95% CI)	*P*-value^*^	Coefficient (95% CI)	*P*-value^*^
Age (years)	−0.004 (−0.090, 0.083)	0.933	—	—
Female gender	−0.007 (−0.043, 0.072)	0.570	—	—
Axial length (mm)	0.252 (−0.583, 1.088)	0.547	—	—
Central corneal thickness (μm)	−0.013 (−0.018, 0.002)	0.112	−0.010 (−0.018, 0.003)	0.135
Corneal curvature (D)	−0.037 (−0.267, 0.194)	0.749	−0.041 (−0.274, 0.192)	0.725
IOP (mmHg)	0.049 (−0.038, 0.136)	0.262	0.058 (−0.033, 0.148)	0.208
PPA_+BM_ width (μm)	−0.000081 (−0.001, 0.001)	0.896	−0.000085 (−0.001, 0.001)	0.892
PPA_−BM_ (γ zone PPA) width (μm)	0.002 (0.001, 0.002)	**<0.001**	0.002 (0.001, 0.002)	**<0.001**
LC thickness (μm)	−0.000283 (−0.006, 0.005)	0.922	−0.001 (−0.007, 0.005)	0.835
Anterior laminar depth (μm)	0.002 (0.001, 0.004)	**0.031**	0.003 (0.001, 0.005)	**0.016**
FoBMO angle (°)	0.039 (−0.026, 0.104)	0.232	0.037 (−0.030, 0.104)	0.274
LC defect	0.723 (0.314, 1.132)	**0.001**	0.720 (0.307, 1.132)	**0.001**
BMO-MRW (μm)				
Global	−0.008 (−0.013, −0.004)	**<0.001**	−0.008 (−0.013, −0.004)	**<0.001**
Temporal-superior	−0.005 (−0.009, −0.001)	**0.019**	−0.005 (−0.009, −0.001)	**0.019**
Temporal	−0.004 (−0.011, 0.002)	0.172	−0.004 (−0.011, 0.002)	0.176
Temporal-inferior	−0.007 (−0.010, −0.003)	**<0.001**	−0.007 (−0.010, −0.003)	**<0.001**
Nasal-inferior	−0.008 (−0.012, −0.005)	**<0.001**	−0.008 (−0.012, −0.005)	**<0.001**
Nasal	−0.006 (−0.009, −0.003)	**<0.001**	−0.006 (−0.009, −0.003)	**<0.001**
Nasal-superior	−0.003 (−0.006, 0.001)	**0.025**	−0.003 (−0.006, −0.0004)	**0.024**
Peripapillary choroidal thickness (μm)				
Global	−0.005 (−0.010, −0.0003)	**0.034**	−0.005 (−0.010, −0.0002)	**0.040**
Temporal-superior	−0.003 (−0.007, 0.001)	0.185	−0.003 (−0.006, 0.001)	0.208
Temporal	−0.004 (−0.008, 0.000)	0.077	−0.004 (−0.008, 0.001)	0.093
Temporal-inferior	−0.006 (−0.011, −0.001)	**0.016**	−0.006 (−0.011. −0.001)	**0.017**
Nasal-inferior	−0.006 (−0.011, −0.002)	**0.010**	−0.006 (−0.011, −0.001)	**0.013**
Nasal	−0.005 (−0.009, −0.0003)	**0.034**	−0.005 (−0.009, −0.0002)	**0.038**
Nasal-superior	−0.003 (−0.007, 0.001)	0.134	−0.003 (−0.007, 0.001)	0.141
Subfoveal choroidal thickness (μm)	−0.002 (−0.004, 0.001)	0.169	−0.002 (−0.004, 0.001)	0.204

CI = confidence interval; D = diopter; IOP = intraocular pressure; BMO = Bruch’s membrane opening; PPA_+BM_ = β-parapapillary atrophy with Bruch’s membrane; PPA_−BM_ = β-parapapillary atrophy without Bruch’s membrane; LC = lamina cribrosa; FoBMO angle = angle of fovea-to-BMO-center axis relative to the horizontal axis of the image frame; MRW = minimum rim width.

Factors with statistical significance are shown in boldface.

^*^P values were adjusted with Benjamini-Hochberg procedure.

In this study, LC defects were identified in 20 (37.74%) of the 53 myopic eyes. Notably, we found that the presence of LC defects was significantly associated with a larger BMO area (*P* = 0.001). Table 3 describes the features of the eyes with LC defects, compared with the eyes without LC defects. Eyes with LC defects had a higher IOP (*P* = 0.006), larger BMO area (*P* = 0.003), larger PPA_−BM_ width (*P* = 0.004), deeper anterior laminar depth (*P* = 0.020), and thinner global and temporal-inferior, nasal-inferior, and nasal BMO-MRW (*P* = 0.003, *P* = 0.003, *P* = 0.005, and *P* = 0.003, respectively) than did eyes without LC defects. Multivariate logistic regression analysis was conducted to identify factors associated with LC defects in myopic eyes with axial lengths between 26 and 27 mm. In order to avoid issues of multicollinearity, BMO area, PPA_−BM_ width, and global BMO-MRW were included in the multivariable model separately, since they significantly associated with one another and showed high variance inflation factors. In the multivariate logistic regression analysis, higher IOP, larger BMO area, and thinner global BMO-MRW were associated with the presence of LC defects in myopic eyes (Table 4).

**Table 3:** Comparison between eyes with or without lamina cribrosa defect.

Variables	Eyes with LC defect (n = 20)	Eyes without LC defect (n = 33)	*P*-value
Age (years)	26.23 ± 1.24	27.00 ± 2.86	0.272
Male, n (%)	14 (70.00)	21 (63.64)	0.768
SE refractive error (D)	−5.96 ± 1.51	−5.41 ± 1.85	0.271
Axial length (mm)	26.44 ± 0.25	26.42 ± 0.28	0.812
Central corneal thickness (μm)	548.75 ± 28.93	555.39 ± 26.97	0.402
Corneal curvature (D)	42.59 ± 1.13	42.56 ± 1.03	0.928
IOP (mmHg)	15.50 ± 2.45	13.39 ± 2.50	**0.006**
BMO area (mm^2^)	3.04 ± 0.86	2.31 ± 0.62	**0.003**
PPA_+BM_ width (μm)	213.00 ± 182.56	155.64 ± 177.12	0.264
PPA_−BM_ (γ zone PPA) width (μm)	507.95 ± 286.20	285.48 ± 190.80	**0.004**
LC thickness (μm)	216.95 ± 48.06	216.55 ± 32.74	0.971
Anterior laminar depth (μm)	380.70 ± 131.23	300.48 ± 80.57	**0.020**
FoBMO angle (°)	−5.14 ± 3.23	−5.65 ± 3.50	0.598
BMO-MRW (μm)			
Global	316.65 ± 34.15	350.27 ± 46.22	**0.003**
Temporal-superior	324.05 ± 37.50	347.85 ± 56.91	0.103
Temporal	235.05 ± 28.21	246.24 ± 36.48	0.246
Temporal-inferior	327.80 ± 56.08	372.64 ± 48.61	**0.003**
Nasal-inferior	364.00 ± 49.90	405.21 ± 49.62	**0.005**
Nasal	340.30 ± 54.11	390.42 ± 59.64	**0.003**
Nasal-superior	368.30 ± 66.52	399.27 ± 85.94	0.174
Peripapillary choroidal thickness (μm)			
Global	137.79 ± 41.02	152.12 ± 48.98	0.287
Temporal-superior	146.74 ± 51.91	163.03 ± 59.35	0.324
Temporal	123.47 ± 37.08	143.09 ± 59.30	0.149
Temporal-inferior	112.32 ± 32.50	125.45 ± 47.47	0.290
Nasal-inferior	118.95 ± 40.81	129.58 ± 45.20	0.402
Nasal	155.00 ± 51.84	165.09 ± 49.31	0.489
Nasal-superior	157.95 ± 56.48	175.33 ± 55.81	0.287
Subfoveal choroidal thickness (μm)	233.85 ± 65.31	260.52 ± 91.60	0.223

D = diopter; IOP = intraocular pressure; BMO = Bruch’s membrane opening; PPA_+BM_ = β-parapapillary atrophy with Bruch’s membrane; PPA_−BM_ = β-parapapillary atrophy without Bruch’s membrane; LC = lamina cribrosa; FoBMO angle = angle of fovea-to-BMO-center axis relative to the horizontal axis of the image frame; MRW = minimum rim width.

Data are mean ± standard deviation unless otherwise indicated.

Factors with statistical significance are shown in boldface.

**Table 4:** Factors associating with lamina cribrosa defect in highly myopic eyes (26 mm ≤ axial length **<**27 mm).

Variables	Univariate		Multivariate (Model 1)		Multivariate (Model 2)		Multivariate (Model 3)	
	Odds Ratio (95% CI)	*P*-value	Odds Ratio (95% CI)	*P*-value	Odds Ratio (95% CI)	*P*-value	Odds Ratio (95% CI)	*P*-value
IOP	1.406 (1.075, 1.839)	0.013	1.315 (1.006, 1.719)	**0.045**	1.301 (1.004, 1.699)	**0.046**	1.506 (1.123, 2.020)	**0.006**
Anterior laminar depth	1.008 (1.001, 1.015)	0.019	1.005 (0.997, 1.014)	0.184	1.004 (0.997, 1.012)	0.264	1.004 (0.996, 1.011)	0.314
BMO area	3.752 (1.548, 9.090)	0.003	2.728 (1.119, 6.653)	**0.017**				
PPA_−BM_ width	1.004 (1.001, 1.007)	0.006			1.003 (0.999, 1.006)	0.163		
Global BMO-MRW	0.978 (0.961, 0.995)	0.012					0.973 (0.953, 0.993)	**0.010**

CI = confidence interval; IOP = intraocular pressure; BMO = Bruch’s membrane opening; PPA_−BM_ = β-parapapillary atrophy without Bruch’s membrane; MRW = minimum rim width.

Factors with statistical significance are shown in boldface.

*P* values were adjusted with Benjamini-Hochberg procedure.

## Discussion

Recent studies have suggested that BMO would be a good anatomical landmark for assessing ONH in myopic eyes, and BMO-MRW measurement based on the BMO has emerged as a promising option in diagnosing myopic glaucoma^11–13,28^. However, how BMO is affected by axial elongation has been determined in less detail, and to the best of our knowledge, no other studies have thoroughly investigated the implication of BMO enlargement on the myopic ONH. In this study, we found that BMO area tended to increase with axial elongation. However the correlation between BMO area and axial length was relatively weak. Interestingly, we identified a notable variation in BMO area among otherwise healthy myopic eyes, and this suggested that BMO enlargement does not necessarily occur in all myopic eyes. BMO enlargement was associated with PPA_−BM_ width, BMO-MRW, and pCT thinning at the temporal-inferior, nasal-inferior, and nasal sectors, as well as the presence of LC defects. The current study findings are important because they indicate that physicians should be aware of sectorial BMO-MRW thinning in myopic eyes with large BMO when evaluating patients with glaucoma, and it might help better understand the pathogenesis of myopic ONH deformations.

Jonas *et al*.^29^ described the myopia-associated widening of BMO around the ONH in their histomorphometric study. The studies performed on normal Japanese and Australian populations reported a significant positive correlation between BMO area and axial length^14,30^. Nakanishi *et al*.^15^ also showed that BMO area was significantly associated with axial length. Our findings are consistent with those of previous reports, and indicate that BMO might undergo morphological changes during axial elongation. Whereas, another study on a normal Caucasian population demonstrated that BMO area was unrelated to axial length. However, that study might not be compared to ours because of the different inclusion criteria; they included only eyes with SE refractive error within −6 D and excluded highly myopic eyes^16^. In a recent prospective longitudinal evaluation of childhood myopia, Kim *et al*.^17^ found that BMO remained stable during myopia progression. However, their observation period was relatively short (2 years). Additionally, a careful assessment of their result showed a tendency for BMO distance to increase as time progressed, even though it did not reach the level of statistical significance (*P* = 0.100).

Previous studies have shown that BM thickness and length in the macular lesion were not affected by axial elongation^31,32^. It has been suggested that the axial elongation-related increase in optic disc-fovea distance was the result of the development and enlargement of the parapapillary gamma zone (in other words, PPA_−BM_ in this study) and that macular BM neither expands nor gets thinner^31,32^. It might be due to the non elastic and relatively high biomechanical strength of BM. Thus, the mechanical strain within the BM during axial elongation might cause the enlargement of BMO, that is the physiological hole, instead of BM stretching.

In this study, a negative correlation was observed between BMO-MRW and BMO area. The dependency of BMO-MRW on BMO size is well known^6,14,15^. With a larger BMO area, the axons of RGCs spread over an increased perimeter, and therefore, the measured BMO-MRW seems to be diminished while still possessing the same number of axons. Interestingly, we found that the association between BMO-MRW and BMO area differed sectorially, with a strong correlation in the temporal-inferior, nasal-inferior, and nasal sectors and a modest correlation in the temporal-superior and nasal-superior BMO-MRW sectors. No correlation was found in the temporal sector. Our results might indicate that BMO enlargement occurs asymmetrically, mainly at the inferior and nasal lesions in myopic eyes. Although the statistical significance was weak, the significant correlations between pCT and BMO area at the corresponding sectors might support our speculation. Therefore, we hypothesize that the temporal site of BMO remains constant, and the enlargement occurs more predominantly at the inferior and nasal sectors of the BMO during axial elongation. Future research that measures BM thickness around the ONH might help understand BMO changes during myopia progression.

In myopic eyes with axial lengths between 26 and 27 mm, pCT was thinnest at the temporal-inferior sector. Our finding agrees with those of previous reports^33,34^. Gupta *et al*.^33^ explored the profile of the peripapillary choroid and found that it was thinnest inferiorly in both myopic and emmetropic eyes. Importantly, the temporal-inferior peripapillary choroid became significantly thinner as BMO enlarged in this study. Given that vascular support for the prelaminar portion of the optic disc originates from the peripapillary choroid^35^, choroidal thinning might be linked to vascular insufficiency, contributing to the increased glaucoma susceptibility in the temporal-inferior region. However, it remains unknown whether the development of myopic glaucoma in the temporal-inferior region is more prevalent in eyes with enlarged BMO than in eyes without enlarged BMO. Future longitudinal studies would be needed to clarify this question.

Because BMO size is not consistent among individuals and shows large between-subject variability in the normal population^14,16^, one may argue that the large BMO found in this study might not be the result of axial elongation-associated ONH deformation, and might just be an inborn characteristic. A limitation of the current study is that our findings are the result of cross-sectional comparisons among myopic eyes, and we did not observe the eyes longitudinally. However, we found a significant positive correlation between PPA_−BM_ width and BMO area after adjusting for age, sex, and axial length. Previous clinical and histomorphometric studies have revealed that PPA_−BM_ (sometimes referred to as gamma zone PPA) width increased greatly with increasing axial length, and PPA_−BM_ is thought to be the result of scleral stretching associated with eyeball elongation^36,37^. Therefore, we suggest that the eyes with large BMO described in our study might have undergone more mechanical strain on the optic disc during axial elongation, and longer PPA_−BM_ width might be an indirect evidence of BMO enlargement.

The LC of the ONH has been proposed as the primary site for glaucoma pathogenesis^38^. Among the various morphological changes found in patients with glaucoma, posterior displacement of the LC is considered a key feature of glaucomatous optic nerve damage. Few studies have conducted detailed investigations of LC depth in healthy myopic eyes^39^. In a study performed on a Korean population, Yun *et al*.^39^ compared LC depth in different axial length subgroups and reported that LC depth did not differ significantly in normal eyes. However, they used BMO as a reference plane, and did not consider the morphological changes of the myopic ONH. In the current study, we measured LC depth relative to the sclerochoroid junction to eliminate the influence of choroidal thickness and found that anterior laminar depth was significantly associated with BMO area. Our finding might indicate that BMO enlargement also accompanies the backward movement of the LC.

LC defects have been extensively studied with respect to their potential as a marker for progressive damage to RGCs in myopic glaucoma^25,40–42^. Han *et al*.^40^ previously described that, even among subjects without glaucoma, LC defects were found in 27.8% of myopic eyes. As in their reports, the current study also found LC defects in highly myopic eyes without any clinical sign of glaucoma and that LC defects were relatively common (37.74%). Notably, BMO area was an important parameter in LC defect formation. We propose that mechanical strain on the LC during BMO enlargement might cause LC defects.

Additionally, IOP was significantly associated with LC defects in highly myopic eyes. In our earlier study, we demonstrated a significant association between IOP and optic disc rotation^42^. In that study, we hypothesized that IOP may result in additional stress on the posterior sclera during axial elongation and might therefore affect the development of myopic ONH deformation. Similarly, IOP-related stress within the connective tissues of the LC might play a role in LC defects. Since the level of IOP-related stress for a given level of IOP is principally determined by the geometry of the load-bearing tissues of the ONH^43^, our hypothesis does not seem unreasonable, even though all subjects in this study had normal IOP.

The strength of this study is the inclusion of young, healthy subjects with high myopia and no ocular disease, who were thus free of confounding factors. However, our study also had some limitations. First, we excluded eyes if BMO was not clearly detectable on 4 or more consecutive B-scan images. There is a possibility of exclusion of potential study subjects that might have influenced the results of our study; thus, some of the parameters may be underestimated. In a recent study, Zheng *et al*.^44^ reported that 28.0% of highly myopic eyes showed indiscernible BMO commonly over the superotemporal and inferotemporal meridians. We also found that a significant proportion of highly myopic eyes had indiscernible BMO over at least 1 meridian. Nevertheless, to minimize the exclusion of potential highly myopic subjects, the exclusion criteria were relaxed in this study, under the assumption that BMO is not a tortuous line but a closed curve with a smooth contour. As a result, only about 6% of the eyes were excluded. Therefore, the effect that the excluded subjects might have had would be negligible. Second, the cause-and-effect relationship between BMO enlargement and significantly associated factors cannot be ascertained owing to the cross-sectional nature of our study. Third, because our study subjects were Korean, the current findings may not be generalized to other ethnic populations. Fourth, we did not evaluate the location and number of LC defects. Future analyses using more detailed information of LC defects may provide additional insights.

In conclusion, BMO area varied among the highly myopic eyes having similar axial lengths. Although our data do not present the underlying mechanism, BMO seems to be enlarged more prominently at the inferior and nasal sectors. Clinicians should be aware of BMO-MRW thinning at the temporal-inferior, nasal-inferior, and nasal sectors when evaluating myopic eyes. Additionally, we clearly observed that the highly myopic eyes with enlarged BMO exhibited characteristic morphologic features of peripapillary choroidal thinning, posterior displacement of the LC, and LC defects. Considering their significant association with glaucoma, our results are important because they highlight the importance of BMO in myopic eyes. Nevertheless, the role of BMO enlargement in the pathogenesis of myopic glaucoma and its value as a predictor of future damage remains to be determined.

## Supplementary information

Supplementary information

## Data Availability

The datasets generated during the current study are available from the corresponding author on reasonable request.
