# Supplementary material for: Bruch’s membrane opening enlargement and its implication on the myopic optic nerve head
Source: Sci Rep. 2019 Dec 20;9:19564. doi: 10.1038/s41598-019-55926-w (PMC6925273; doi:10.1038/s41598-019-55926-w)

**Bruch’s membrane opening enlargement and its implication**

**on the myopic optic nerve head**

Mi Sun Sung,^1^ Min Young Heo,^1^ Hwan Heo,^1^ and Sang Woo Park^1*^

^1^ Department of Ophthalmology and Research Institute of Medical Sciences, Chonnam National University Medical School and Hospital, Gwangju, South Korea

**Supplementary Figure S1.** Example of focal LC defect. (A) Fundus photography (B) Infrared fundus image. The green arrow shows the cross-sectional location of the B-scan. (C) EDI OCT B-scan image. B-scan image demonstrates defect (yellow arrowhead) at the temporal far periphery of the LC, adjacent to the LC insertion.


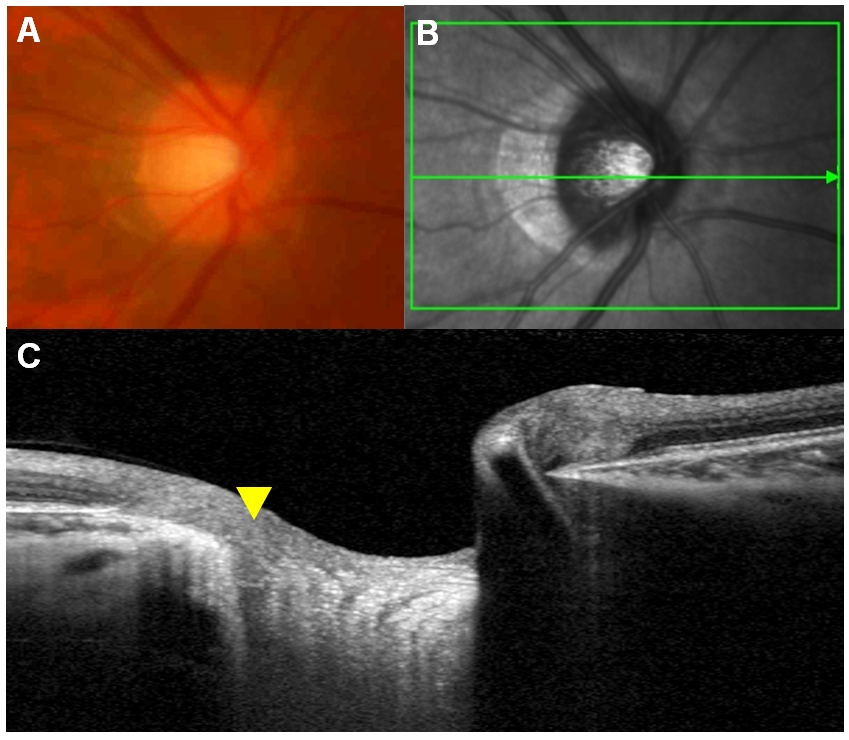

Supplement: Supplementary file 1 — Supplementary information [file 41598_2019_55926_MOESM1_ESM.docx]
